# Durability of Forging Tools Used in the Hot Closed Die Forging Process—A Review

**DOI:** 10.3390/ma17225407

**Published:** 2024-11-05

**Authors:** Grzegorz Ficak, Aneta Łukaszek-Sołek, Marek Hawryluk

**Affiliations:** 1 GK FORGE, Przemysłowa 10 Street, 43-440 Goleszów, Poland; gficak@gkforge.pl; 2 Faculty of Metals Engineering and Industrial Computer Science, AGH University of Krakow, Av. Mickiewicza 30, 30-059 Krakow, Poland; alukasze@metal.agh.edu.pl; 3 Department of Metal Forming, Welding and Metrology, Wroclaw University of Science and Technology, Lukasiewicza Street 5, 50-370 Wroclaw, Poland

**Keywords:** hot die forging, destructive mechanisms, tribology, lubrication, tool steel, numerical calculations, artificial neural networks, wear

## Abstract

The article presents the classification of the wear mechanisms of forging tools. The durability of dies can be enhanced through a variety of methods, including the selection of appropriate hot working tool steel, the application of effective heat treatment, the utilization of advanced surface engineering techniques, and the incorporation of lubricating and cooling agents. Two popular methods of tool regeneration, such as re-profiling and laser regeneration, are presented. The issue of numerical wear prediction based on the Archard model, the correlation of this model with experimental results, low-cycle fatigue (HTLCF), and an alternative method based on artificial neural networks are discussed. The paper aims to present currently known wear mechanisms and the methods of increasing and predicting tool durability.

## 1. Introduction

In the current industrial era, the methods of manufacturing metal components are extremely diverse, ranging from modern machining technologies to traditional forging processes. ranging from modern machining technologies (e.g., additive manufacturing) [1,2]. Among the various techniques employed, the use of forged products continues to be regarded as a highly esteemed solution, primarily due to their exceptional strength and durability. Forging technology, despite some limitations, continues to play a key role in the manufacturing of metal components, especially where high mechanical strength and precise forming are required [3,4]. However, the use of forging technology is not free from its challenges, especially in terms of production costs. Forging tools are a significant component of the total cost of the manufacturing process. The estimated cost of tools, given their durability and complexity, can significantly affect the economics of production. Therefore, there is an ongoing need to understand and optimize the costs associated with forging technology, encompassing both the cost of the tool itself and the costs associated with its maintenance and replacement [3,5,6].

**Figure 1:**
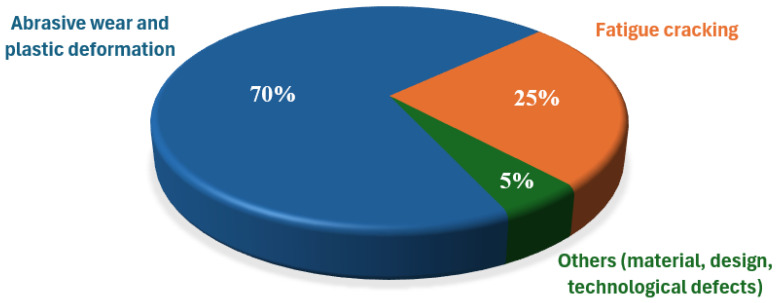
Contributions of individual factors that reduce hot forging tool life [7].

The wear mechanisms of forging tools are a complex research area that deals with analyzing the various processes and factors that lead to tool degradation (Figure 1). This process is influenced by the properties of the tool materials (e.g., strength, hardness, fatigue life, etc.) and the working conditions under which the tools are used (e.g., forging temperature, loads, and others). This process is influenced by the properties of the tool materials and the working conditions under which the tools are used. Understanding these mechanisms is key to optimizing the manufacturing process, improving tool life, and reducing costs associated with tool maintenance and replacement. Forging tool life is understood as the number of parts produced with a given tool. With reduced die life, production costs can increase by up to 40%. Wear mechanisms have a significant impact on their durability, as it is estimated that 70% of tools are withdrawn due to plastic deformation or abrasive wear [7,8,9,10].

Due to the extreme environment in which hot working tools operate, no single wear mechanism can be assigned to the entire die cavity area. Examples of these mechanisms were discussed in [7,11].

Abrasive wear, in which the material is gradually eroded due to friction. In the surface contact areas, abrasive particles, both hardened and loose, act as micro-sharpeners, leading to the gradual removal of the material. To minimize the process, it is essential that the surfaces are smoothed thoroughly to a high standard and that lubricants are employed regularly to reduce friction and prevent excessive material wear. This type of wear is also affected by scale. If left in the die cavity, it intensifies abrasive wear, resulting in the formation of grooves [11]. Plastic deformation, mainly observed in die cavities and punches, results from the tempering process, which leads to a reduction in flow stress. This in turn, under pressure, leads to the plastic deformation of the tools. By carrying out the heat treatment properly, it is possible to increase the hardness of the dies and the yield strength of the material. However, it is necessary to maintain a balance between resistance to the tempering process and resistance to fracture [12]. Tool thermal fatigue is a phenomenon that arises due to the occurrence of cyclical temperature fluctuations at the surface layer of the tool. Due to the limited thermal conductivity of the material, a temperature gradient occurs between the core and the tool surface. As a result, the temperature fluctuations are accompanied by the alternating compression and tension of the material leading to the formation of micro-cracks. This phenomenon, which results from the interaction of thermal and mechanical fatigue mechanisms, is referred to as thermo-mechanical fatigue. Adhesive wear mainly affects the top layer of tools, characterized by a localized increase in roughness. As a result of metallic bonding and frictional forces, the material is torn out or smeared on the tool surface. Adhesive wear can be reduced by surface quality. The better the surface quality of the tools and the smoother the transitions (radii), the less chance of oxidation or adhesion occurring. Fatigue cracking results from the accumulation of elastic deformation in the surface layer, which leads to a local loss of material cohesion. Repeated stress loading results in increased stresses in these areas, initiating the formation of micro-cracks. In further operation, these cracks propagate, leading to tool failure. This type of wear occurs primarily in areas of high-stress concentration, such as rounding radii, grooves, or holes [13]. Oxidation, which involves the formation of oxides in the surface layer of tools, is the result of oxygen diffusion in micro-areas that have been deformed both plastically and elastically. The separation of oxide layers occurs as a result of the action of frictional forces. For wear by oxidation to occur, the rate at which oxide layers are formed must exceed the rate of abrasion.

Hot forging tools are exposed to extreme conditions that require high strength, high temperature resistance, and minimal deformation during operation. In response to these challenges, engineers and scientists are developing a variety of methods to increase the durability of these tools to ensure their efficient and safe use in forging processes. Currently, there is no single proper method for increasing the durability of tools. It is influenced by the mechanisms previously described, the design of the dies, the heat treatment, and the lubrication method; therefore, each forging process must be considered individually [9]. 

The main aim of our work is to present a review of our research and that of others that concerns currently used methods of increasing and predicting tool durability used in the forging process at elevated temperatures.

## 2. Methods to Increase Tool Life

Based on literature research and our own research, it can be concluded that currently, there are only general guidelines and directions covering the search for effective methods or ways of increasing the durability of forging tools. At the same time, it is important to treat and analyze each process individually because many technological parameters and tribological conditions are closely related to the process and strictly dedicated to a given process. Sometimes even a small change in these parameters or a slightly different geometry of the forging causes the working conditions of the tools to be completely different. The most popular methods of increasing durability include the selection of the tool material and its appropriate heat treatment, optimization of tool design, as well as methods related to the surface layer (hybrid techniques, thermochemical treatment, welding, and mechanical methods), and other methods not directly related to a tool [14].

### 2.1. Selection of Hot Working Tool Steel

Hot working tool steels used in forging can be divided into three groups. The first group includes steels used for inserts, press dies, and die casting molds. These tool steels are usually subjected to long contact times between the hot billet and the die cavity. The second group of hot tool steels is characterized by short charge contact with the working area, dynamic working, and high single-stroke pressures. Those steels are primarily used for dies for hammer forging. The third group comprises steels for rolls, as referenced in [15,16,17,18]. The most commonly used steels for hot working in the forging industry are 1.2713, 1.2714, 1.2343, and 1.2344 according to DIN norms. These steels are distinguished by their favorable mechanical properties and wear resistance. To achieve optimal properties, they are tempered two or three times at the temperature of approximately 500 °C, thereby capitalizing on the secondary hardness effect.

In addition to the conventional hot working tool steels employed in the forging industry, steels produced by Voestalpine AG (Linz, Austria), including the Unimax, Vidar Superior, Dievar, Skolvar, and Orvar Supreme varieties, are currently available on the market. The Unimax and Vidar Superior steels undergo electro-slag remelting, which ensures an even distribution of carbide precipitates. This process can also reduce sulfur content, non-metallic inclusions, and segregation. Improving these factors increases the mechanical properties, especially wear resistance [19,20].

**Figure 2:**
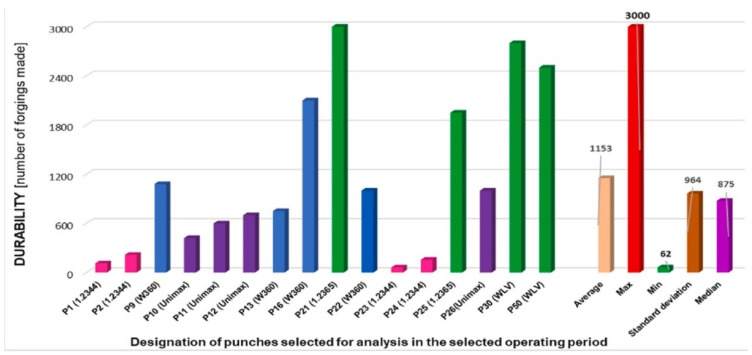
Durability of punches made from different materials [16].

In work [16], the tests of punches made from 4 different materials are presented: W360, 1.2365 (WLV), Unimax, and 1.2344 (WCLV). The correctly selected steel grade was shown to have the most significant influence on tool life (Figure 2), while surface engineering methods did not yield good results [21]. Steel 1.2365 showed high resistance to abrasive wear at elevated temperatures and resistance to plastic deformation. Based on the scanning results, a very intensive loss of calotte height was observed for punches made of 1.2344 and Unimax steel. In contrast, the wear is much slower for punches made of W360 and 1.2365 steel. The enhanced service life of punches produced from material 1.2365 can be attributed to optimal structural fit. It was demonstrated that the reduction in frictional forces occurred in the carbide separation area. For hot work tool steels with carbide inclusions in the microstructure, it is important that the carbides are evenly distributed throughout the whole volume and do not form carbide clusters, and that they do not surround the grains at their boundaries, as this arrangement reduces both strength and impact strength, and generally the service life [16].

In the research study presented in work [22], an attempt was made to replace the use of tungsten with vanadium. During the tests, it was observed that the material exhibited higher hardness in the “red hardness” state, which then decreased during subsequent heating. This phenomenon was attributed to the separation of M6C carbide particles between 10 nm and 400 nm in size. In the tempered state, the material displayed a tensile strength of 1792 MPa, a total elongation of 21.4%, and a yield strength of 1338 MPa. Other research on a new hot working tool steel has been proposed in paper [23]. In it, RATE steels are presented, which are characterized by the fact that they exhibit a strengthening phenomenon in the ‘hot work hardened state’. The main consideration of these steels is the low temperature of the α → γ transformation (AC1 temperature is about 600 °C) and the high stability of the overcooled austenite. The mentioned steels are readily chip-machinable, which is attributable to their ferritic structure. The cost of the steel is relatively low, a consequence of its classification as a medium alloy steel and the fact that the amount of alloying elements does not exceed 10%. It has also been shown that repeated deformation of the steel at the temperature of 450 °C provides significant hardening of the RATE steel—from 180 to 460 MPa—and the degree of hardening was maintained until heating to the temperature of 750 °C. As a result, there is an increase in die strength during service [23]. In RATE steels, dispersion and nanophase hardening utilize the presence of dispersed particles or nanoparticles in the material structure to provide strengthening. Polymorphic transformation hardening is defined as the set of structural modifications that are induced in a material as a consequence of its transformation from one crystalline phase to another. In addition, strain hardening in the two-phase (γ + α) region is based on the increase in strength through the deformation of one phase by the other and their interaction [23,24].

**Figure 3:**
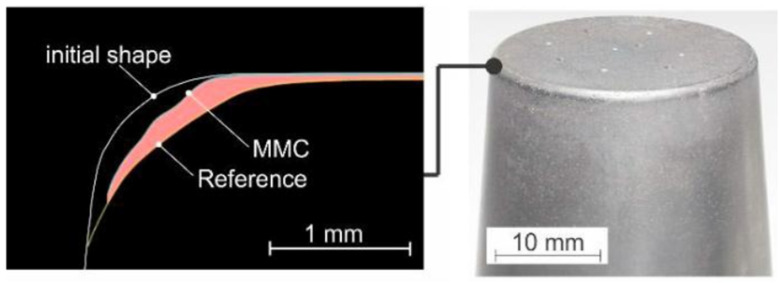
Comparison of the initial radius of the punch and the profile of worn punches after 100 cycles [25].

A different group of materials that can find applications as a hot working material are metallic matrix composites (MMC). Paper [25] presents a tool produced by sintering and forging. In it, 1.2344 steel powder was combined with fused tungsten carbide (FTC) particles to create the tool. Stamps were made from the resulting material, and tests were carried out. The profile of the MMC stamp was observed to exhibit a greater degree of convexity and concavity compared to the reference sample (Figure 3). 

**Figure 4:**
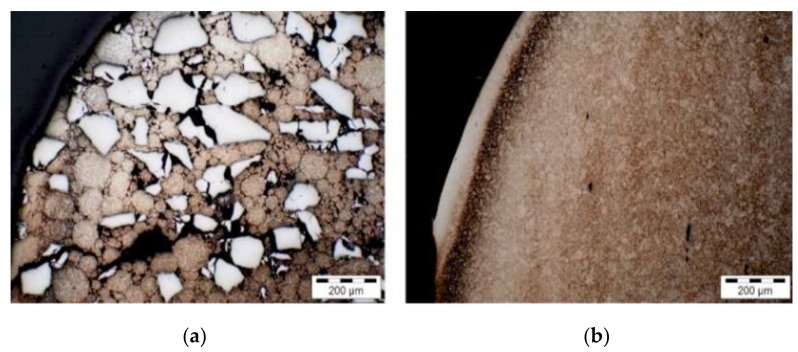
Metallography of punches after 100 cycles: (**a**) MMC composite, (**b**) reference test [25].

The profile shape was influenced by the FTC particles, which protected the material against abrasion (Figure 4). Such tools represent an alternative concept for extending tool life.

Paper [26] presents the fabrication process of H13 steel enriched with trace TiC nanoparticles. It was found that the ceramic nanoparticles promote martensitic transformation, serving as nucleation sites for austenite. The produced steel exhibited higher yield strength, tensile strength, and impact strength. During the testing phase, it was determined that the material exhibited a 28.6% enhancement in wear resistance and a 40.4% improvement in surface roughness.

### 2.2. Heat Treatment of Hot Working Tool Steels

**Figure 5:**
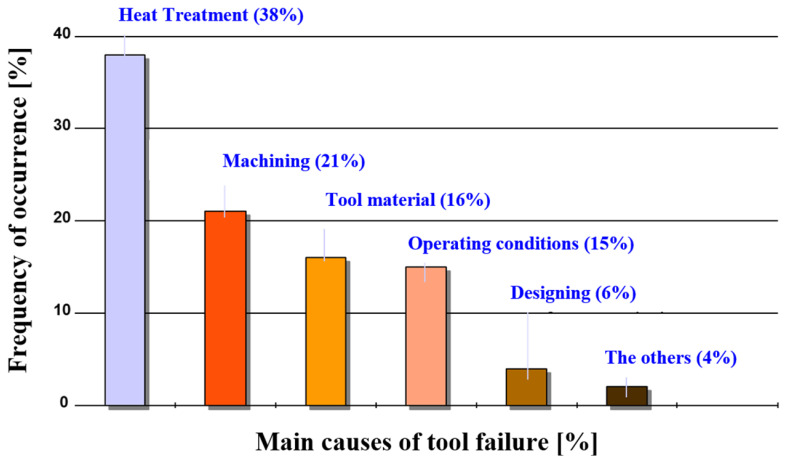
Causes of sudden failures of forging tools [27].

In the manufacturing of forging tools, especially those designed for hot working, the heat treatment of steel plays a key role. Heat processes have a significant effect on the mechanical properties, strength, and dimensional stability of tools, which has a direct impact on the quality of the components produced. Properly selected heat treatment can increase tool life and enable tools to be used effectively under extreme operating conditions. As indicated by [27], most early tool failures can be attributed to improper heat treatment, accounting for approximately 38% of cases (Figure 5).

**Figure 6:**
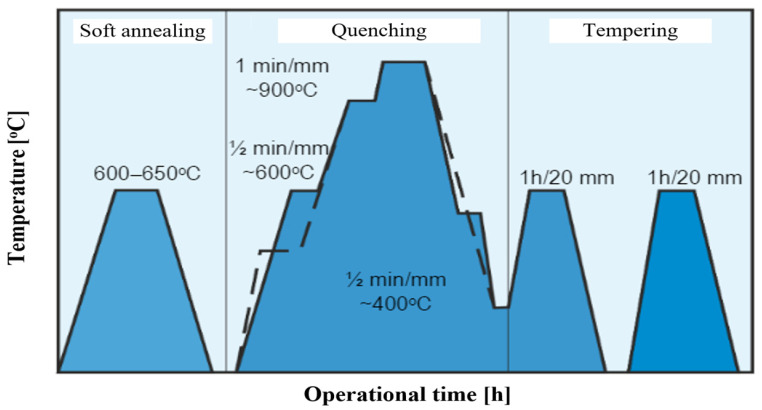
Heat treatment process of hot working tool steel [34].

Correct heat treatment of hot working tool steels should consist of annealing, quenching, and two or three times tempering [28] (Figure 6). Repeated tempering results in an increase in notch strength but a decrease in yield strength and tensile strength [29,30,31,32,33]. In each case, the temperature required to austenitize a particular grade of steel has been selected to avoid the excessive growth of residual austenite. The cooling of the tools usually takes place in oil (less frequently in the air), e.g., oil OH-70. Subsequently, repeated tempering is carried out to facilitate the transformation of the residual austenite. The tempering temperature is usually higher than the working temperature of the tools. The structure after correct heat treatment should consist of tempered martensite and finely dispersed, evenly distributed carbides [3,27,28,34,35].

A replacement for the classical heat treatment of H11 steel was proposed in work [36], namely intermittent martensitic hardening and bainitizing (iQ&B). The structure after such treatment consists of tempered martensite, residual austenite, bainitic ferrite, and carbides [37]. Lukasiewicz et al. [36] demonstrated that a one-step iQ&B treatment results in a significant enhancement in uniform elongation, exhibiting an increase of approximately 2.5–3.4 times and a notable rise in impact strength, with a 1.7–2.9 time increase compared to conventional heat treatment. This observed improvement can be attributed to the elevated level of residual austenite susceptible to the TRIP effect and the presence of a fragmented bainitic structure. Studies on the effects of tempering and cryogenic treatment are presented in [38,39,40,41]. Cryogenic treatment was proven to increase hardness and wear resistance. The wear rate decreased by 59% for the HTT_500_CT_200_ process (double tempering at the temperature of 500 °C, cryogenic treatment, tempering at the temperature of 200 °C) [38]. The implementation of HTTCT manufacturing techniques has been demonstrated to reduce surface roughness, thereby limiting the susceptibility for crack initiation and propagation. When cryogenic treatment is applied, there is a reduction in residual austenite after heat treatment. On the other hand, the use of nitriding allows favorable compressive stresses to be generated in this layer, which increases the service life [39].

**Figure 7:**
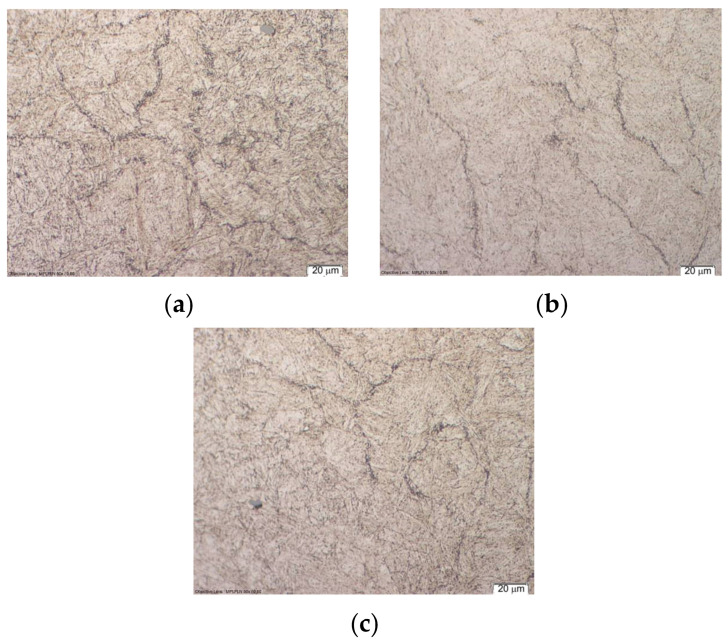
Microstructure of the die insert made of X40CrMoV5-1 steel observed in the (**a**) XY plane, (**b**) ZX plane, and (**c**) ZY plane [42].

The impact of inappropriate heat treatment is presented in work [42]. It was noted that during heat treatment, carbides were formed at the boundaries of the former austenite to facilitate crack propagation. In the bright areas, conglomerates of non-metallic inclusions were located (Figure 7).

These are sulfides, oxides, and nitrides, which contribute to crack nucleation. The reason for this phenomenon was the inappropriate tempering temperature (450–500 °C), i.e., up to the secondary hardness peak. This resulted in a decrease in the impact strength of the material.

### 2.3. Surface Engineering

Surface engineering plays a key role in all areas of industry, but particularly in forging tool manufacturing. The surface of forging tools significantly impacts their performance, durability, and the quality of the parts produced. In recent years, there have been significant developments in surface engineering technologies and methods to improve the performance properties of forging tools. The topic of surface engineering of forging tools has been repeatedly analyzed by many researchers. In articles [7,43,44,45,46,47], the following methods of increasing the durability of surface layers are presented: nitriding, pad welding, mechanical treatment, and duplex coating. Nitriding increases hardness and resistance to plastic deformation and improves corrosion and oxidation resistance. Mechanical treatment improves the mechanical properties of the surface and generates residual stresses in metals. A distinction is made between nitriding [48], nitrocarburizing, surfacing, sol–gel coatings [49], CVD coatings, PVD, and hybrid methods [50], e.g., CVD-coated nitriding or gas nitriding with a hybrid layer consisting of a nitride layer combined with a Cr/CrN coating [51]. In addition to the mentioned methods, several coating applications and thermo-chemical treatments (including plasma nitriding) were also examined [52,53,54]. Furthermore, an investigation was conducted to assess the effect of protective coatings on the interaction between tool steel and the workpiece material [55]. Additionally, NITREG nitriding was also explored [56]. 

Particularly noteworthy are surface-hardening laser methods due to the advantages of the process, i.e., minimal deformation of the workpiece material and very good surface roughness, which eliminates finishing, and local strengthening of the surface layer [57,58]. Laser methods can also be used to apply coatings.

Paper [59] presents the laser deposition of Stellite 21 coating on H13 tool steel. Stellite 21 coatings are cobalt–chromium–tungsten-based alloys with a small amount of carbon, and I use them mainly to improve wear resistance.

In addition to methods and ways to increase the durability of the top layer of tools, research has been carried out on the influence of the phase structure of nitrides on tool durability. In paper [60], it was shown that using the ZeroFlow method of nitriding in a single-component NH3 atmosphere, a surface layer with different nitride phase structures could be obtained. The susceptibility of γ′ nitrides to fracture in regular lattice as well as the susceptibility of γ′ nitrides to spallation in regular lattice were demonstrated. Layers with a composite zone of the ε phase were characterized by high abrasion resistance. For the nitride layer without composite zones (α-type), good thermo-mechanical strength was observed.

In the study presented in [61], the deposition of TiCrN/DLC coatings from the vaporous phase through cathodic arc deposition (CAPVD) was presented. High abrasion resistance, low coefficient of friction provided by the DLC layer, and high cohesion of the coating with the substrate were demonstrated. It was proven that wear resistance did not increase with coating thickness by the fact that in the study the best properties were obtained with a coating thickness of 3 µm (coatings of 2 to 6 µm were applied at an interval of 1 µm). The introduction of a DLC layer on top of the TiCrN coating was shown to increase wear resistance. The piston tool coated with this layer had twice the tool life of the uncoated tool.

One of the key determinants in understanding the durability of a surface layer is its roughness. Conventional methods such as lapping, grinding, and polishing are lengthy and mostly manual. An alternative technology to these methods is burnishing. A significant advantage of the process is that the material is not removed but instead is burnished, which results in the generation of compressive stresses [62,63]. In the case of the fast laser deposition process (EHLA), the surface roughness is influenced by the size of the powder particles and the deposition rate (best results are achieved if the rate does not exceed 70%) [64,65].

### 2.4. Lubricants and Cooling Agents

The prevailing wear mechanism, as evidenced by the statistical analysis, is abrasive wear [7,8,9]. Lubricants and coolants are used to reduce this mechanism. Graphite lubricants, graphite-free lubricants, water–oil emulsions, and cooling gasses are available on the market. In Polish forging companies, they are mainly applied to surfaces by spraying using compressed air spraying equipment. Lubricants consist of lubricating particles, surface-active additives, and binding agents that facilitate the distribution and formation of lubricating films on the surface of the die cavity [66,67].

**Figure 8:**
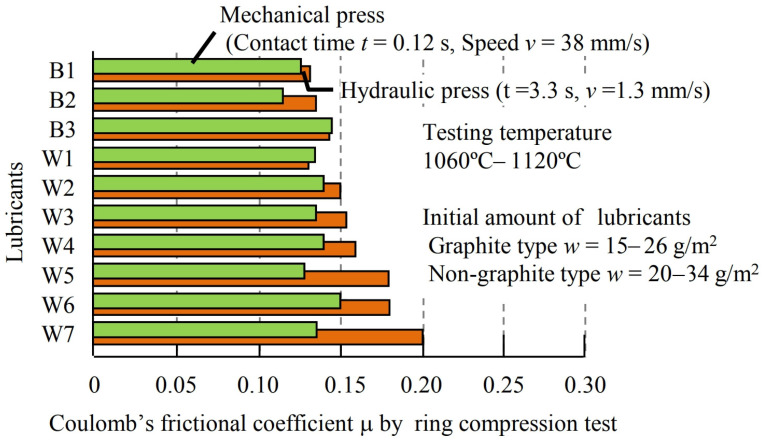
Coefficients of friction determined in the ring compression test for various lubricants using both a mechanical press and a hydraulic press. B1, B2, and B3 denote graphite-based lubricants, while W1–W7 denote non-graphite-based lubricants [68].

Asai and Kitamura [50] carried out ring compression tests on three graphite mixtures and seven graphite-free mixtures. For the experiment, they used a mechanical press for testing with an average compression rate of v = 38 mm/s and a compression time of t = 0.12 s and a hydraulic press with v = 1.3 mm/s and t = 3.3 s (Figure 8).

It was shown that in the case of a mechanical press, the Coulomb’s friction coefficient ranged from 0.12 to 0.15. In contrast, the experiment performed on a hydraulic press showed higher friction values with lubricants of the non-graphite types.

**Figure 9:**
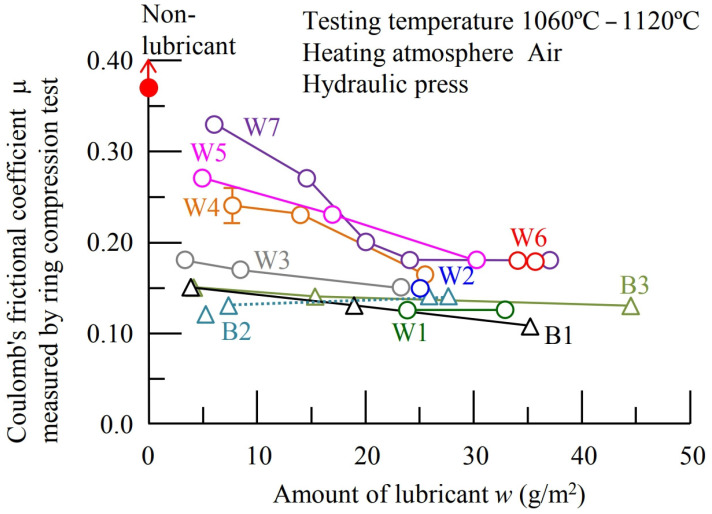
The influence of lubricant quantity on the friction coefficient [68].

They also conducted a study to determine the effect of the amount of lubricant–coolant on the coefficient of friction at low compression rates. It was shown that graphite lubricants maintained low and stable friction coefficient values when the amount of lubricant was low (Figure 9). 

The heat transfer to the tools was investigated in work [69]. For this purpose, an oil-based grease was used, which was a mixture of soybean oil and graphite powder in a ratio of 1:1. Deltaforge3 was used as the water-based graphite grease, with a mixture ratio of water and graphite powder of 3:1. It was shown that the water-based lubricant adhered correctly to the surface in the temperature range of 150–350 °C, while for the oil-based lubricants, correct bonding occurred from the temperature of 250 °C to the temperature of 850 °C. Also, temperature tests were carried out on the stamp surface. Oil-based lubricants showed a higher thermal conductivity. This is because the oil fills the roughness irregularities and voids between the tool and the workpiece material, whereas with water-based lubricants, solid graphite remained at the phase boundaries, creating local voids where air was present to limit heat transfer. It was also determined that the coefficient of friction on oil-based lubricants was 0.3 and on water-based lubricants 0.35 [69]. In work [70], they conducted a study to determine the convective heat transfer coefficients for a graphite grease (FB400) and an emulsion (Berulit625). They showed that the cooling performance improves with increasing surface temperature. This phenomenon can be attributed to the formation of a protective vapor mantle around the surface, which serves to insulate the material. The vapor mantle, in turn, exhibits high thermal resistance, which results in a reduction in thermal conductivity. As the vapor sheath cools down, a stable vapor sheath layer cannot be maintained. This is related to the Leidenfrost temperature [70].

**Figure 10:**
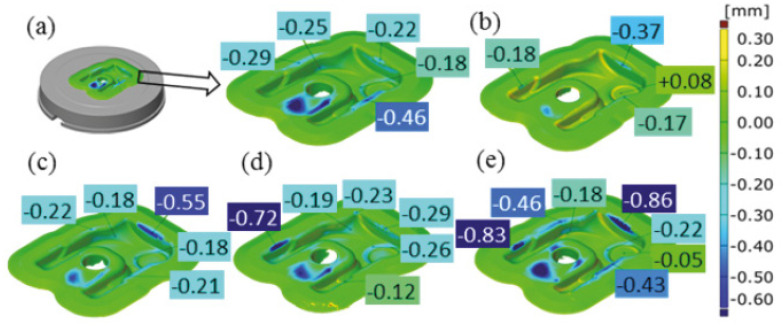
Comparison of scanning results of die inserts: (**a**) X37CrMoV5 steel—manual lubrication after 8000 pcs; (**b**) X37CrMoV5-1 steel—automatic lubrication after 9000 pcs; (**c**) UNIMAX steel—automatic lubrication after 8000 pcs; (**d**) after 16,000 pcs; (**e**) UNIMAX steel—manual lubrication [72].

In addition to the correct choice of lubricant/coolant, tool life is also affected by how tools are lubricated. Articles [27,71,72,73] demonstrated that the optimal spray angle and direction impact the uniformity of tool wear. Additionally, a relocation of the wear areas was observed (Figure 10). Furthermore, it has been demonstrated that an inappropriate lubricant–coolant ratio can contribute to the formation of micro-cracks and unfilled areas [74]. The reason for this is the local increase in pressure caused by air pockets. 

## 3. Reconditioning of Tools

**Figure 11:**
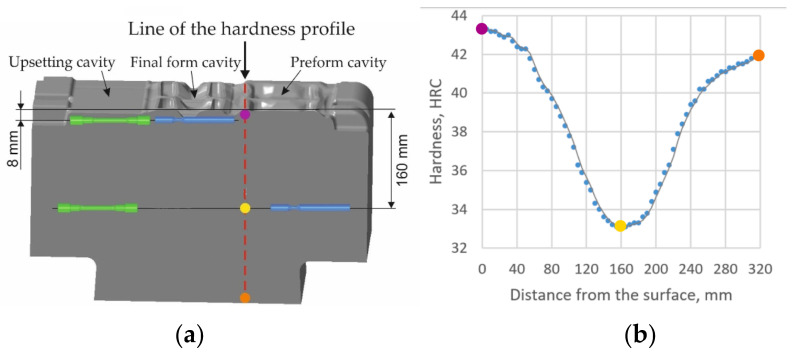
View of the (**a**) location of the sample taken from 56NiCrMoV7 steel, (**b**) hardness profile of the extracted sample [75].

The most common method of forging tool reconditioning is reprofiling. It involves lowering the entire die cavity by the damaged layer. As a result, the profile of the die cavity remains constant while the size of the tool is reduced. The main disadvantage of this solution is the limited number of reprofiles and the fact that there is a limited depth of improvement of the material on the through-hole. As a result, the newly obtained surface layer has lower strength properties than the original layer. A critical number of regenerations, after which heat treatment must be applied, was also determined. This value is not constant for each tool, as the hardness gradient at the tool cross-section will always be different [75] (Figure 11).

Another method for remanufacturing tools—laser remanufacturing—was presented in the works [76,77]. This method consists of adding molten material to worn parts using a laser beam. The regeneration process consists of three stages: surface cleaning, DED or PBF surfacing, and post-processing. Compared to traditional surfacing, it provides a smaller heat-affected zone, fewer localized distortions, and cracks [78,79,80]. Research on laser reconditioning of tools made of H13 steel was presented in paper [81]. It was shown that the LMD-deposited area had a higher wear resistance than the substrate, which was mainly due to the presence of martensite and carbides in the matrix.

Due to the numerous disadvantages of laser remanufacturing, such as shape inaccuracy, surface cracking, or porosity, laser deposition is combined with post-processing processes such as heat treatment, machining, shot blasting, etc. A commercial hybrid laser remanufacturing system combined with turning and milling has been proposed by DG Mori. A similar solution has been proposed in works [82,83,84]. The main disadvantage of the quoted solutions is the reduced fatigue resistance due to porosity being a crack initiator [76]. 

## 4. Tool Life Prediction

Forecasting forging tool life is an important issue in manufacturing engineering. Through the use of appropriate methods and analytical tools, it is possible to predict when a tool will require maintenance, repair, or replacement. As a result, this enables production processes to be optimized, minimizing machine downtime and reducing tool maintenance costs.

### 4.1. Numerical Simulations of Durability and Wear

Currently, there are many forging process simulation programs on the market, including QForm UK, Forge, Abaqus, and DeForm. In addition to analyzing the correctness of the process, it is possible to estimate tool wear [85,86,87]. For tool life prediction, the Archard model is most often chosen [88,89,90], by which wear can be estimated to be directly proportional to normal pressure and slip rate and inversely proportional to hardness. The authors determined the areas most susceptible to wear. As a result of their research, they developed tools with the lowest predicted wear.

(1) $$Wear=K\int \frac{p^{a}v^{b}}{H^{C}}$$

where p—normal pressure, v—velocity, H—hardness, and a,b,c, *K*—constants that depend on the co-working materials.

**Figure 12:**
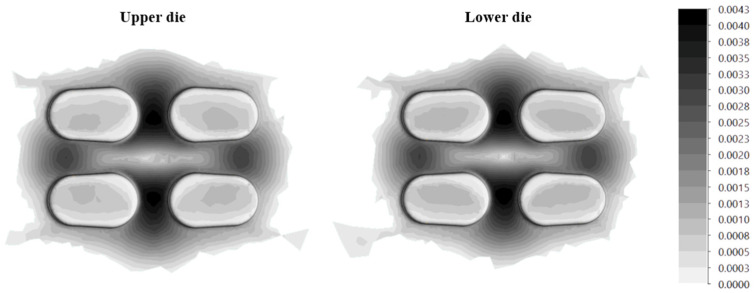
Distribution of the wear coefficient of preliminary forging dies due to normal pressure actions [91].

A simulation using the Archard model is presented in work [88], where the wear was predicted (Figure 12) for both forging dies (upper and lower).

The wear of tools was calculated using the following equation:

(2) $$W_{p}=\int_{0}^{t}\frac{K_{p}*p^{a}*V_{\tau }^{b}}{\bar{\sigma^{a}}}\;dt$$

where $W_{p}$—tool wear as a result of normal pressures, $K_{p}$—empirical coefficient of wear pressure, a and b—Bayer_exp_a and Bayer_exp_b empirical coefficients, p—normal pressure at the point of contact between the material and the tool, expressed in MPa, t—tool-to-material contact time in seconds, $\bar{\sigma }$—yield strength of the material expressed in MPa, and $V_{\tau }$—the tangential velocity of the workpiece contact node relative to the tool, expressed in m/s.

Lee and Jou [92] have assumed that the consumption coefficients are not constant in the Archard model. They proposed the following equation:

(3) $$W\left(T\right)=k\left(T\right)\frac{LP}{H\left(T\right)}$$

where $k\left(T\right)$—consumption coefficient as a function of temperature and $H\left(T\right)$—hardness as a function of temperature 

Further research on the Archard model was presented in paper [93]. Particular attention was paid to the fact that the Archard model was effective for predicting abrasive wear. The downside is that it does not take into account other wear mechanisms. Therefore, the authors proposed their model (Z), which, in addition to the Archard model (W), takes into account wear resulting from the other mechanisms F(N,T).

(4) Z=W+F(N,T)

**Figure 13:**
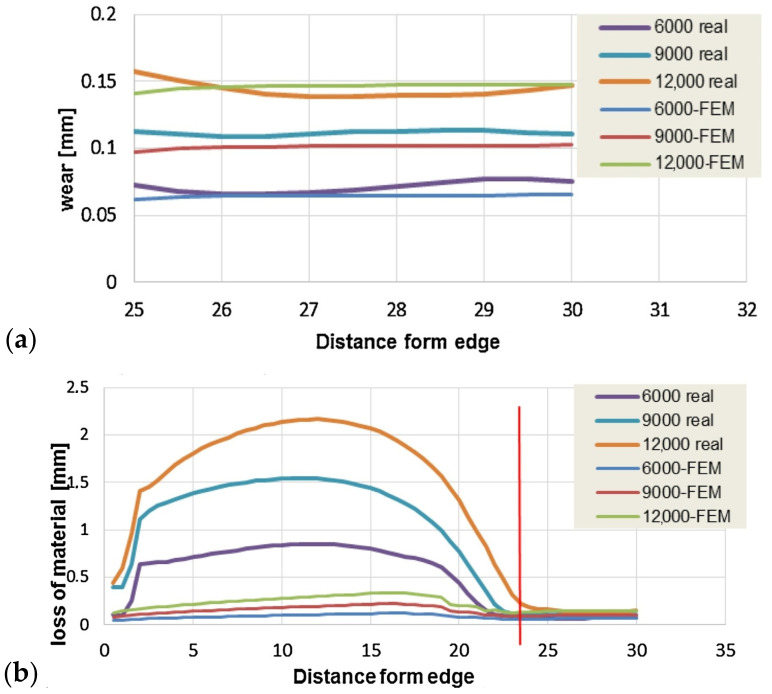
Comparison of predicted wear using the Archard model with the experimental results: (**a**) 100% simulation–experiment agreement. (**b**) 20% simulation–experiment agreement [93].

Numerical simulation was also carried out using Marc Mentat 2013.1 software. (Hexagon, Stockholm, Sweden). Areas were determined where the proportion of abrasive wear was almost 100%, so the Archard model would have been sufficient. Areas where abrasive wear accounted for only 20% of the total wear were also determined. In these areas, thermomechanical wear predominated. During the tests, three stages of wear were delineated on the graph (Figure 13), and the consumption curve resembles the classic (Lorentz) [93].

A different approach to tool life simulation was proposed in work [94], where the authors focused on high-temperature low-cycle fatigue (HTLCF). Abaqus software and the implemented Darveaux model were used to predict wear. In this model, the fatigue life corresponds to the total number of fatigue crack initiation cycles and fatigue crack development cycles.

(5) $$N_{0}=C_{1}∆W^{C_{2}}$$

where $N_{0}$—number of cycles required to initiate fatigue damage, ∆W—cumulative inelastic hysteresis energy, $C_{1}$ and $C_{2}$—material constants.

Propagation prediction was implemented using the following equation:

(6) $$\frac{dD}{dN}=\frac{C_{3}}{L}∆W^{C_{4}}$$

where D—damage parameter, D—number of cycles, $C_{3}$ and $C_{4}$—material constants calculated concerning the element size of the FE model, L—the characteristic length of the element.

For ductile materials, it was assumed that the reduction in material strength was modeled using the scalar damage parameter D. For each cycle, the stress tensor was calculated as follows:

(7) $$\sigma =(1-D)\bar{\sigma }$$

where σ—stress tensor of the damaged material, $\bar{\sigma }$—tensor of the undamaged material, D—damage parameter.

Finally, the modeling and crack initiation equations were determined:

(8) $$N_{f}=N_{0}+N_{\alpha }$$

(9) $$N_{\alpha }=\frac{D}{dD/dN}$$

where $N_{f}$—total number of cycles, $N_{\alpha }$—number of damage propagation cycles.

As a result of the tests, two areas of maximum stress were determined. It was estimated that as a result of continuous cyclic loading, or more specifically, after 2200 parts had been produced, a 2 mm thick crack would form in the tools [94].

Xu, Li, and Wang, while examining the surface using electron microscopy, found that the wear of the pivot occurs through adhesive wear, abrasive wear, and oxidation [95]. Based on the experiment, they proposed a wear model that accounts for the mechanisms:

(10) $$W_{total}=\lambda_{adhesive}*w_{adhesive}+\lambda_{abrasive}*w_{abrasive}+\lambda_{oxidation}*w_{oxidation}$$

(11) $$w_{adhesive}=\int_{0}^{t}k\frac{P*V}{H\left(T\right)}\mathrm{d}t$$

(12) $$w_{abrasive}=\int_{0}^{t}\frac{2\tan\theta }{\pi }\frac{P*V}{H\left(T\right)}\mathrm{d}t$$

(13) $$w_{oxidation}=\int_{0}^{t}\frac{PA_{P}C^{2}}{H\left(T\right)\xi }\cdot \exp\left(-\frac{Q_{p}}{RT}\right)\mathrm{d}t$$

where $W_{total}$—the total wear depth of the worn surface; $w_{adhesive}$, $w_{abrasive,}$ $w_{oxidation}$—the wear depth, $\lambda_{adhesive}$, $\lambda_{abrasive}$, $\lambda_{oxidation}$—the weighting factors of the individual wear mechanisms.

To determine the weighting factors, wear tests were carried out using the HSR-2M machine. Then, a change in mandrel geometry was performed based on the proposed wear calculation model, numerical forging simulation, BP neural network, and SQP algorithm. The results of the wear simulation and the experimental tests carried out showed good correlation [95].

**Figure 14:**
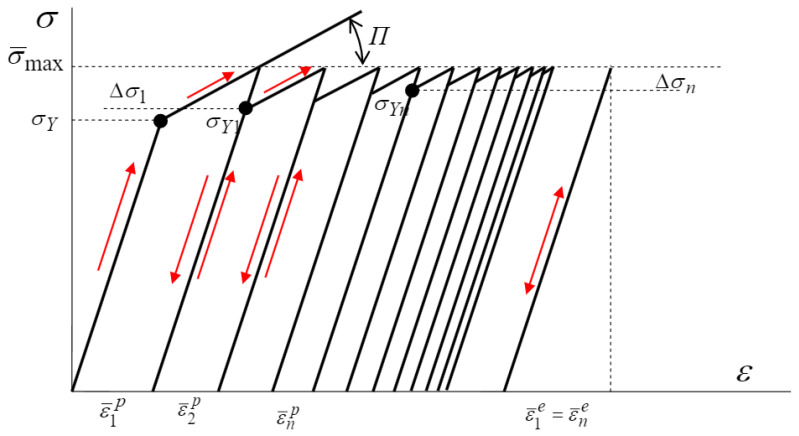
Accumulation of plastic deformation under cyclic loading of dies considering material softening [96].

The low cycle fatigue (LCF) model has been implemented in a number of studies, as outlined in the works of [96,97,98]. In the model, the loading of the dies was assumed to be a combination of elastic-plastic loading and elastic-only loading. The sum of plastic and elastic deformation damage was summed according to the deformation-kinetic failure criterion, and elastic deformation damage was calculated using the elastic component of the Manson–Coffin–Basquin equation. Plastic deformation accumulation was assumed to depend on the thermocycle softening mechanism and a reduction in yield stress proportional to plastic deformation over the cycle. The accumulation and plastic strain model based on linear quenching used in the model is shown in Figure 14, and the total number of cycles to crack formation was calculated using the following formula:

(14) $$N_{f}=\frac{1-{\left(\frac{{\bar{\varepsilon }}_{\Sigma }^{p}}{\varepsilon_{cr}}\right)}^{a}}{2{\left(\frac{{\mathrm{\sigma ’}}_{f}-\sigma_{qm}}{\sigma_{qa}}\right)}^{\frac{1}{b}}}$$

where ${\bar{\varepsilon }}_{\Sigma }^{p}$—total plastic deformation, $\varepsilon_{cr}$—critical deformation, a—exponents reflecting the non-linearity of damage accumulation, *b*—fatigue strength exponent, $\sigma^{\prime }f$—fatigue strength factor, $\sigma_{qm}$—average cycle stress, $\sigma_{qa}$—amplitude of cycle stress

In summary, one of the key limitations of the numerical wear analysis of forging tools is that the wear models implemented in FEM are mostly based on the extended Archard model, which mainly describes abrasive wear well. In contrast, the other main wear mechanisms are much less well described. It is difficult to incorporate all (at least the key) wear models into a single global model in the FEM. Another important limitation is the inclusion of work cycles in the numerical modeling, as a given mechanism in the industrial process occurs at different times and with varying intensity and can introduce interactions.

### 4.2. Predicting Tool Durability Based on Neural Networks

Tool life can be determined using artificial neural networks. Such an attempt was presented in work [99]. The developed system worked with a pooled database of tools—mainly punches used in the pre-die-making operation. The network learning dataset had 750 records determined from experimental studies. Thanks to this study, a system was developed to assess the percentage of individual destructive mechanisms.

Hawryluk and Mrzygłód [100] developed a tool life prediction system (SEPEK-2) using artificial neural networks. Three different hot forging processes were used to build the knowledge module. An interesting result was obtained with the SEPEK-2 tool. It was revealed that during the hot forming process for nitrided, cooled, and lubricated tools, the dominant failure mechanism was thermomechanical fatigue and not abrasive wear. In the case of SEPEK-2, the global error was 10%.

### 4.3. Additive Toolmaking

Nowadays, the industry is becoming more and more convinced to use modern manufacturing technologies to create parts and tools more quickly, precisely, and efficiently. One such innovative method is additive manufacturing, which is also known as 3D printing. Traditionally, forging tools have been manufactured using machining methods; however, additive manufacturing opens up new possibilities in the design and production of these tools [3].

Numerous attempts have been made to produce tools from H10, H11, H13 steels using L—PBF, LMD, DED, and SLM methods. With these methods, the main obstacle encountered is porosity and local remelting defects. These defects can act as crack initiators, especially during thermal fatigue [101,102,103].

In works [104,105,106], the impact of additive manufacturing on product properties was investigated. It was demonstrated that the fatigue life is twice as low as that observed in the conventional process (studies for H13 steel). The main reasons are LOF defects (lack of remelting) and residual porosity. It was also shown that the direction of the structure influences the anisotropy of the fatigue properties.

Heat treatment is used to improve the properties of additively manufactured tools. H13 steel after AM has a cellular-dendritic structure with a tendency to segregate heavy alloying elements. It has been found that direct tempering after AM leads to the decomposition of residual austenite and the precipitation of secondary carbides above the temperatures of 550 °C [107]. If tempering is carried out in the temperature range of 600–700 °C, the interdendritic areas dissolve, hardness decreases, and microstructural changes occur, contributing to rapid wear and brittle fracture [108].

## 5. Summary

The article presents a classification of currently known wear mechanisms and methods for die repair. Based on the comprehensive analysis conducted in this area, supported by our own research, the following most important conclusions can be drawn:

- Two primary methods of refurbishment are distinguished. The first is re-profiling, which is the fastest and therefore the least expensive when considering only the aspect of tooling refurbishment. The main drawback of this method is that the newly formed surface layer possesses lower strength properties compared to the original layer, and the number of possible refurbishments is limited [75]. The second group of refurbishment methods involves cladding, which is carried out using powder wires and additive techniques such as Directed Energy Deposition (DED) and Powder Bed Fusion (PBF). The primary drawbacks of additive solutions include residual porosity and lack of fusion (LOF), both of which reduce the durability of the tooling compared to that of newly manufactured equipment [78,81].
- The discussion of methods aimed at increasing tool durability has been addressed in the context of the appropriate selection of tool steel, heat treatment processes, and lubricating-cooling agents. Numerous researchers focus their studies on the durability of punches, which is a justified approach given that this component of the tooling system typically exhibits the shortest lifespan. Efforts are being made to replace steels such as 1.2343 and 1.2344 with alternatives like Unimax, W360, 1.2365, and RATE steels [16,23]. It has been demonstrated that the appropriate selection of steel significantly increases tool durability. In addition to the use of “conventional” steels, research is ongoing into metal matrix composites. These studies are still in their early stages, as tests have so far been conducted only at the laboratory scale. Nonetheless, the results are highly promising, as the punches exhibited a wavy edge pattern during experiments, attributed to the presence of fused tungsten carbide particles, which protected wear [25].
- The article also discusses coatings that enhance tool durability, with particular emphasis on Stellite 21 coatings, TiCrN coatings deposited from the gas phase using cathodic arc deposition, and the ZeroFlow method, which involves nitriding in a single-component NH3 atmosphere. Researchers highlight the importance of coating thickness, as it has been demonstrated that increased thickness does not necessarily improve wear resistance. Furthermore, it has been shown that the application of DLC layers on top of TiCrN coatings significantly enhances abrasive wear resistance in dies, providing a strong foundation for further research into the use of dual coatings [61].
- The work also presents methods for predicting and assessing tool durability. A significant challenge lies in monitoring the current state of the tool during operation. One potential solution is the reverse scanning method, which involves scanning the forged part and comparing the results to baseline values. Durability prediction is carried out through numerical simulations, with researchers relying on the Archard wear model and extending it by incorporating additional factors. In addition to abrasive wear, they are attempting to implement fatigue wear, adhesive wear, and oxidation into the model. The results have shown a good correlation, particularly for punches [93]. This may be attributed to the fact that the primary wear mechanism for nitrided, cooled, and lubricated tools is not abrasive wear but rather thermomechanical fatigue, as demonstrated using artificial neural networks [100].
- In summary, the durability of hot forging tools is a complex research area that involves the analysis of various processes and factors. The most significant progress in research has been observed in the case of punches. This may be because the abrasive wear mechanism is well understood by researchers, and the lower costs associated with laboratory and experimental studies of punches compared to the entire tooling system. However, there is a subset of tools that are less studied in terms of durability, particularly those where the die cavity is predominantly filled using extrusion methods. Future research should focus on these tools to better understand their wear mechanisms and develop strategies to enhance their longevity.

## Data Availability

No new data were created or analyzed in this study.
